# Defect and Doping Engineered Penta-graphene for Catalysis of Hydrogen Evolution Reaction

**DOI:** 10.1186/s11671-021-03590-3

**Published:** 2021-08-13

**Authors:** Jinbo Hao, Feng Wei, Xinhui Zhang, Long Li, Chunling Zhang, Dan Liang, Xiaoguang Ma, Pengfei Lu

**Affiliations:** 1 School of Science, Xi’an University of Architecture and Technology, Xi’an, 710055 China; 2 State Key Laboratory of Information Photonics and Optical Communications, Beijing University of Posts and Telecommunications, Beijing, 100876 China; 3 School of Physics and Optoelectronic Engineering, Ludong University, Yantai, 264025 China

**Keywords:** Penta-graphene, Hydrogen evolution reaction, Electrocatalysis, First-principles calculation

## Abstract

Water electrolysis is a sustainable and clean method to produce hydrogen fuel via hydrogen evolution reaction (HER). Using stable, effective and low-cost electrocatalysts for HER to substitute expensive noble metals is highly desired. In this paper, by using first-principles calculation, we designed a defect and N-, S-, P-doped penta-graphene (PG) as a two-dimensional (2D) electrocatalyst for HER, and its stability, electronic properties and catalytic performance were investigated. The Gibbs free energy (Δ*G*_H_), which is the best descriptor for the HER, is calculated and optimized, the calculation results show that the Δ*G*_H_ can be 0 eV with C2 vacancies and P doping at C1 active sites, which should be the optimal performance for a HER catalyst. Moreover, we reveal that the larger charge transfer from PG to H, the closer Δ*G*_H_ is to zero according to the calculation of the electron charge density differences and Bader charges analysis. Ulteriorly, we demonstrated that the HER performance prefers the Volmer–Heyrovsky mechanism in this study.

## Background

Because of the climate change and environmental pollution caused by fossil fuels usage, exploitation and utilization of clean and renewable energy are the mean way after nowadays [1–4]. As a clean, renewable and environmentally friendly energy source, hydrogen (H_2_) has been attracting considerable attention to fulfill human future energy needs [5, 6]. Water electrolysis is a sustainable and clean method to produce H_2_, and electrocatalysts can enhance the efficiency of water splitting observably [7, 8]. For hydrogen evolution reaction (HER), platinum-based nanomaterials are considered as the best electrocatalysts because of a small Tafel slope, a low overpotential, a slightly negative Gibbs free energy (Δ*G*_H_) and a high exchange current density [9, 10], but the scarcity and high cost hamper their industrial scale applications [11]. Therefore, developing effective, earth-abundant and low-cost electrocatalysts is essential for HER [12–14].

In fact, a wide range of earth-abundant electrocatalysts have been studied and designed for HER [15–17]. Among these materials, two-dimensional (2D) nanomaterials provide new opportunities for HER because of the compelling structural and electronic properties. To date, the transition metal dichalcogenides (TMDs) and the graphene-based materials are the biggest and most intensively studied groups of 2D electrocatalysts for HER [18–23]. The TMDs HER catalysts have low overpotential and small Tafel slope, unusual electronic properties and high air stability, exhibit high HER performance, and different methods were taken for enhancing their catalytic performance [24, 25]. The graphene-based HER catalysts have attracted considerable attention and persistent studying because of their distinctive structural merits, such as high electrical conductivity, large surface area and good chemical stability [26, 27]. Many methods were taken for enhancing the catalytic activity, such as heteroatom doping and defection engineering [28, 29]. Meanwhile, the intensive research on other new 2D carbon allotropes have also been developed, such as graphdiyne [30] and penta-graphene (PG) [31]. As a 2D carbon allotrope, PG is composed of only carbon pentagons and inherits many exceptional properties of 2D materials, such as finite electronic band gap, abundant active sites and large surface area, so it is anticipated to be a versatile material for lots of potential applications like other 2D graphene-based materials [32–35]. Since there are only applications in gas adsorption [36–38], H_2_ storage [39, 40], anode materials at present [41, 42], no report has ever been found on the application in HER. Therefore, research on HER by PG is of great significance and cannot only fill such a gap but also broaden the scope of graphene-based HER catalysts. However, the pristine PG is found to be inert for the HER with a relatively large Δ*G*_H_, which means that hydrogen adsorption is difficult and inhibits the HER. This is similar to the problems encountered by pristine graphene (Δ*G*_H_ = 1.85 eV [43]). Heteroatom doping into graphene-based materials could adjust their electronic and catalytic properties, which makes them prospective catalysts for the practical applications [3]. Therefore, we managed to tailor the catalytic activity of PG by heteroatom doping [44–46] and defection engineering [47, 48].

In this paper, by using first-principles calculation, we designed and demonstrated a defect and N-, S-, P-doped PG and investigated their stability and electronic properties and evaluated their performance as HER electrocatalysts. Our results reveal that the defect and doped PG can obviously enhance the catalytic activity toward HER, compared with the pristine PG. It is also shown that the Δ*G*_H_ can be 0 eV with C2 vacancies and P doping at C1 active sites, which should be the optimal performance for a HER catalyst, so P-doped PG has the optimal Δ*G*_H_ and activation energy barrier for the rate-determining step among the three counterparts, and it exhibits more favorable performance. We further show that the catalytic activity arises from the incorporated doping atoms, which can provide efficient pathway for charge transport during the electrolysis, resulting in the reduction in Δ*G*_H_. We also demonstrate that the Volmer–Heyrovsky mechanism is more preferred for HER on defect and doped PG. We compared our results with that of other researchers on graphene, and it can be found that the defection and doping engineering are more effective for PG in catalysis of HER. Thus, our effort on defect and doped PG makes it a high promising electrocatalyst for HER, and our findings provide a deep understanding in designing efficient and durable electrocatalysts. This method can be also applied to other graphene-based materials.

## Computational Methods

Our first-principles calculations were performed using the Vienna Ab initio Simulation Package (VASP) [49]. The projected augmented wave (PAW) potentials were used to analyze the interactions between core electrons and valence electrons [50–52]. The electron exchange–correlation interactions were described by using the Perdew–Burke–Ernzerhof (PBE) functional within the generalized gradient approximation (GGA) [53]. The DFT-D3 exchange–correlation functional was introduced in structural optimization to take the van der Waals interaction into account. The vacuum space along the *z*-direction was set to 20 Å in order to eliminate the interactions between PG and its periodic images.

The plane-wave energy cutoff was set to be 500 eV. The convergence criterion was set as 10^−5^ eV for a total energy. All the atomic positions and lattice structures were fully relaxed with the threshold of a maximum force of 0.02 eV Å^−1^. In order to ensure the accuracy and efficiency of the calculation, a Gamma-centered k-point mesh with a Monkhorst–Pack method 5 × 5 × 1 was employed for all considered structures after convergence test [54]. The amount of the charge transfer between the C atoms and H atoms was calculated using Bader code [55]. We also calculated H* adsorption energy barriers using the climbing image-nudged elastic band (CI-NEB) method [56, 57]. The CI-NEB is an efficient method to determine the minimum energy path and saddle points between a given initial and final position [58–60], and in our CI-NEB calculations, the initial and the final structures were fully optimized.

The adsorption energy (Δ*E*_H_) is defined as

$$\Delta E_{{\text{H}}} = E(*{\text{H}}) - E(*) - \frac{1}{2}E({\text{H}}_{2} )$$

where *E*(*H) and *E*(*) are the total energy of structures with and without hydrogen adsorption, respectively, and *E*(H_2_) is the total energy of a H_2_ molecule.

The Gibbs free energy (Δ*G*_H_) is defined as:

$$\Delta G_{{\text{H}}} = \Delta E_{{\text{H}}} + \Delta E_{{{\text{ZPE}}}} - T\Delta S_{{\text{H}}}$$

where Δ*E*_H_ is the adsorption energy, Δ*E*_ZPE_ is the difference in zero-point energy, *T *is the temperature (298.15 K) and Δ*S*_H_ is the entropy difference of H adsorbed and H in the gas phase. We approximated the entropy of hydrogen adsorption as $\Delta S_{{\text{H}}} \approx \frac{1}{2}(S_{{{\text{H}}_{2} }}^{ \circ } )$, where $S_{{{\text{H}}_{2} }}^{ \circ }$ is the entropy of gas phase H_2_ at standard conditions, *T*Δ*S*_H_ was set to be − 0.202 eV after calculation in this study.

## Results and Discussion

### Structure and Catalytic Activity of Defect and Doped PG

**Fig. 1:**
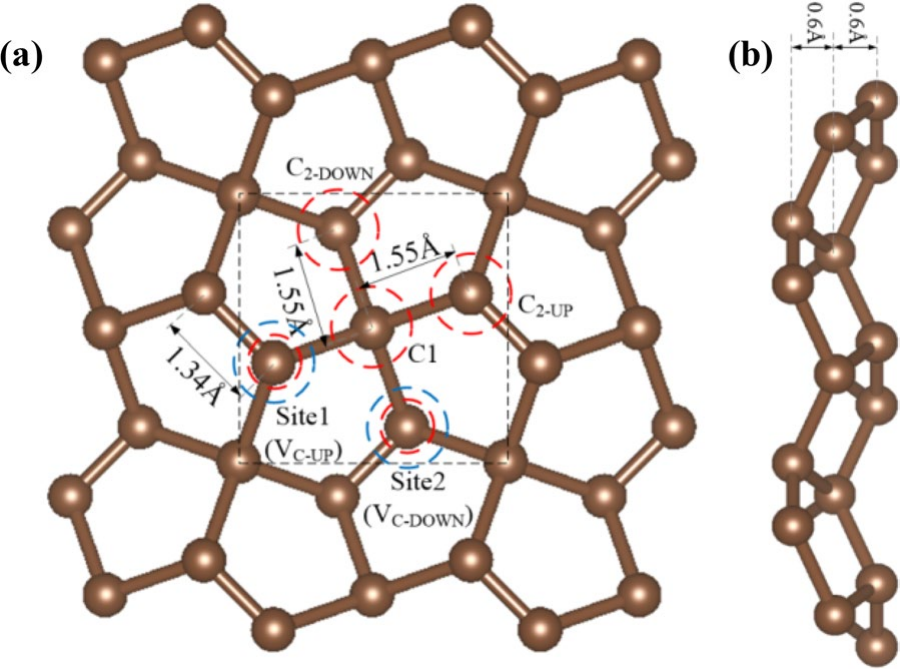
**a** Top and **b** side views of the optimized structure of PG. The black dashed rectangle indicate unit cell, the blue dashed circles indicate two C vacancy sites, the red dashed circles indicate doping sites used in this paper

The optimized structure of PG is shown in Fig. 1. For convenience of discussion, we hereafter group the *sp*^3−^ and *sp*^2−^ hybridized C atoms as C1 and C2, respectively. The distance between the C1 and C2 is 1.55 Å, and the C2–C2 bond length is 1.34 Å, which is consistent with the experimental result [31].

**Fig. 2:**
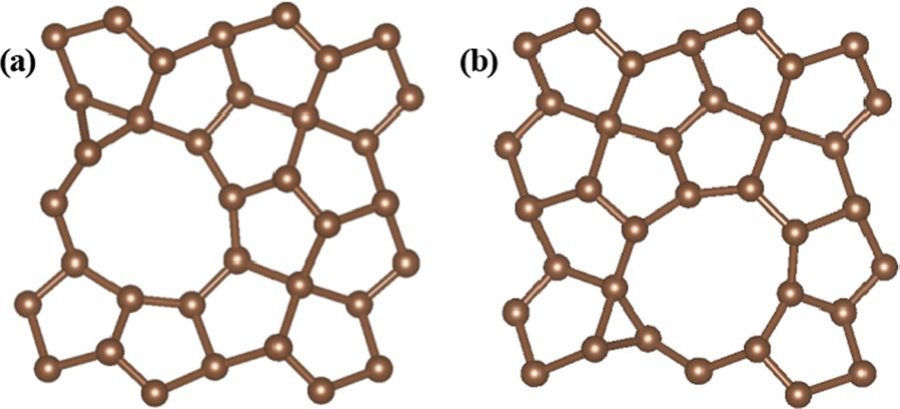
The optimized structures of PG with two different C2 vacancy sites. **a** V_C-UP_ C2 vacancy site, **b** V_C-DOWN_ C2 vacancy site

At the beginning, we first investigated the sites C1 and C2 in the basal plane of pristine PG for HER, the calculated Δ*G*_H_ values are 2.43 eV and 2.72 eV, respectively. So our calculations show that the pristine PG is found to be inert for the HER with a relatively large Δ*G*_H_ of H, which means that hydrogen adsorption is difficult and HER is inhibited. Therefore, we managed to use some methods to tailor the catalytic activity of PG. We researched the possible active sites for doping and we also investigated the active sites for C1 and C2 with N, S, P doping, respectively. The calculation results show that no obvious improvement of HER can be obtained if only doping engineering was introduced. In the case of the P-doped structure, the calculated Δ*G*_H_ values of C1 and C2 sites are 1.24 eV and 1.40 eV, respectively. Ulteriorly, we investigated the defect PG with C vacancy sites. The calculation results reveal that C1 vacancy structure cannot improve the HER performance but C2 vacancy structure can decrease Δ*G*_H_ obviously, so we use C2 vacancy structure in this study. The optimized structures with V_C-UP_ and V_C-DOWN_ C2 vacancies sites are shown in Fig. 2, the vacancy defects are built by removing C2 atoms from C_2-UP_ or C_2-DOWN_ site in a 24-atom supercell. The calculated Δ*G*_H_ values are shown in Table 1, where C1 and C2 are the active sites for hydrogen adsorption.

**Table 1:** Calculated Δ*G*_H_ values for different possible active sites

Structure	Site	Δ*G*_H_ (eV)
Pristine	C1	2.43
	C2	2.72
N-doped	C1	1.48
	C2	1.99
S-doped	C1	1.53
	C2	1.65
P-doped	C1	1.24
	C2	1.40
V_C-UP_	C1	0.24
	C2	0.25
V_C-DOWN_	C1	0.23
	C2	0.24

**Fig. 3:**
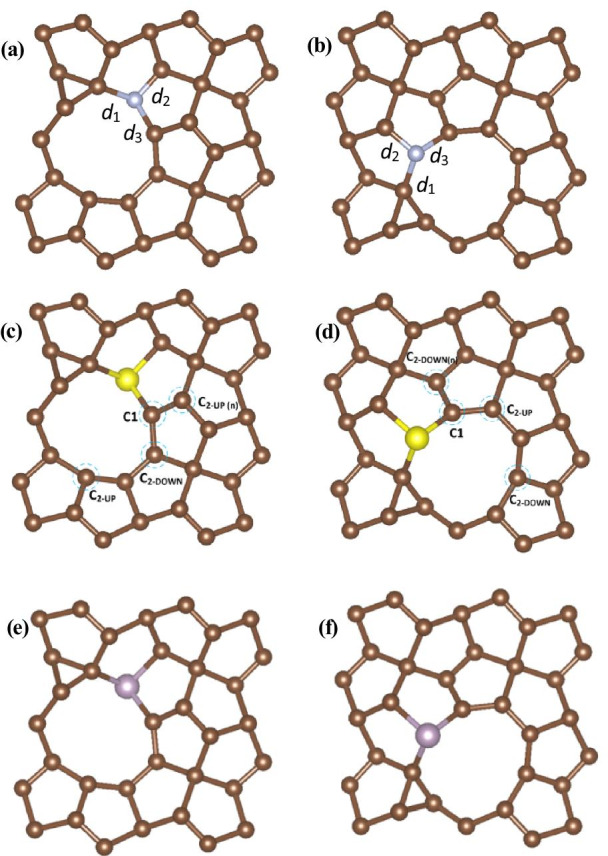
The optimized structures for the defect and doped PG with the two different C2 vacancy sites, **a** V_C-UP_N-doped, **b** V_C-DOWN_N-doped, **c** V_C-UP_S-doped, **d** V_C-DOWN_S-doped, **e** V_C-UP_P-doped, **f** V_C-DOWN_P-doped, the blue dashed circles indicate possible active sites for hydrogen evolution

Though it is confirmed by our calculations that C2 vacancies are efficient to enhance the HER activity, PG with C2 vacancy structure is not yet optimal for a HER catalyst. Thus, we further investigated the defect and doped PG for HER. We used PG with C2 vacancy as initial structure, which is shown in Fig. 2 and then investigated all the different possible active sites with N, S, P doping, including C1, C_2-UP_ and C_2-DOWN_ sites. As a result, we found that better HER performance could be achieved with a combination of C2 vacancy and heteroatom doping. We investigated all the possible structures, and the results showed that there are two structures that can achieve better HER performance, one structure is a combination of the C_2-UP_ vacancy and heteroatom doping in the C_2-DOWN_ site, and the other is a combination of the C_2-DOWN_ vacancy and heteroatom doping in the C_2-UP_ site. So we focused on these two structures and found that they can shift the Δ*G*_H_ values closer to zero. The optimized structures are shown in Fig. 3, and the calculated bond lengths are summarized in Table 2.

**Table 2:** Calculated bond lengths of C2 vacancy and N-, S-, P-doped PG

Structure	*d*_1_ (Å)	*d*_2_ (Å)	*d*_3_ (Å)
V_C-UP_N-doped	1.50	1.39	1.42
V_C-UP_S-doped	1.95	1.79	1.73
V_C-UP_P-doped	1.89	1.81	1.79
V_C-DOWN_N-doped	1.51	1.39	1.42
V_C-DOWN_S-doped	1.95	1.80	1.74
V_C-DOWN_P-doped	1.89	1.81	1.80

We can see that there is slight difference between the corresponding bond lengths of N-doped PG and that of pristine PG. Because of the large radius of S and P atoms, these two structures undergo much more distortion, but they can both maintain the structure of PG.

**Fig. 4:**
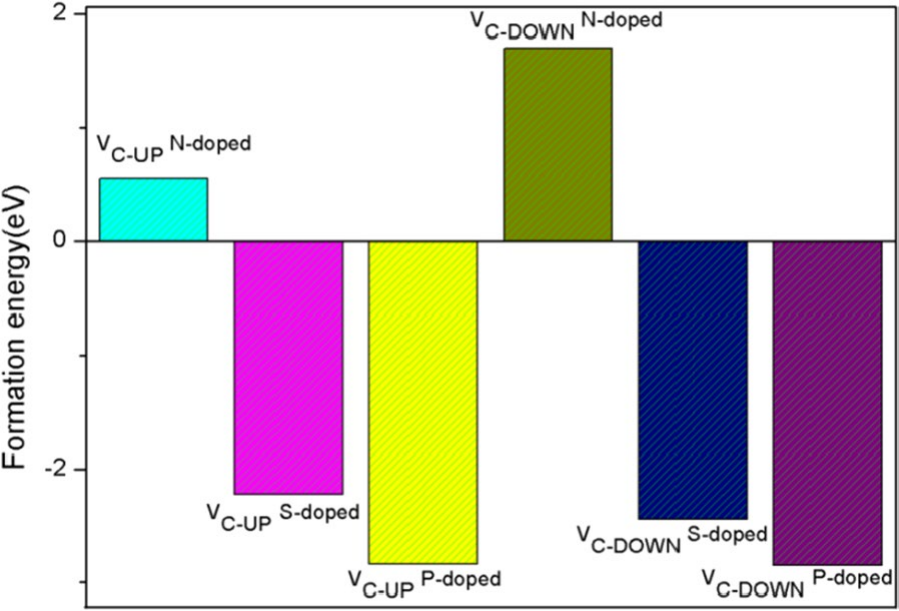
Formation energy of two initial defect and doped PG structures with C1 active sites for HER, more negative value indicates higher stability of the structure

To investigate the stability of PG with C2 vacancy and heteroatom doping, we calculated the formation energy, which is defined as

$$E_{{\text{f}}} = \left( {E_{{\text{t}}} - E_{{\text{V}}} + E_{{\text{C}}} - E_{{\text{d}}} - \frac{1}{2}\mu_{{\text{H}}} } \right)$$

where *E*_t_ is the total energy of the defect and doped system, and *E*_V_ is the energy of C2 vacancy PG, *E*_C_ is the average energy per C atom of the pristine PG, *E*_d_ is the energy of doping atoms,$\mu_{{\text{H}}}$ is taken from the total energy of the H_2_ molecule, respectively. One of our calculation results about the formation energies of preceding two structures with C1 active sites for HER is shown in Fig. 4. We can see that negative formation energies indicate energetically favorable and feasible defect and S-, P-doped PG. Similarly, *E*_f_ values of N-doped structures with active sites for HER are all positive. We investigated all the possible active sites and got the similar results as shown in Fig. 4, so we will investigate only the S- and P-doped PG. According to the definition, a more negative *E*_f_ value indicates higher stability of the structure, so P-doped PG has excellent stability, as well as good HER performance.

### Origin of the HER Catalytic Activity

#### DOS and Band Structures

**Fig. 5:**
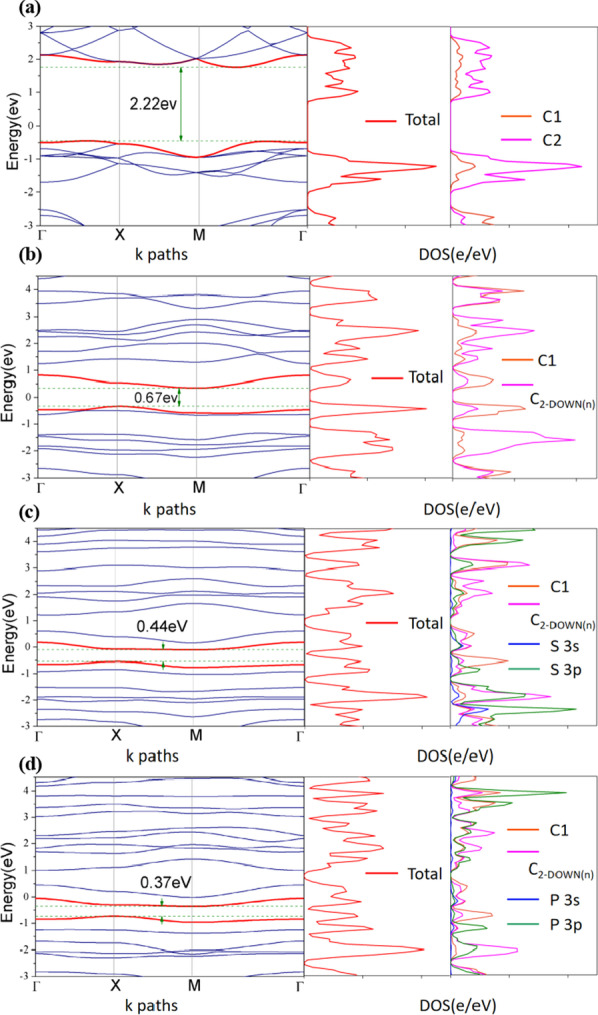
Electronic band structure, total and projected DOS of the defect and doped PG. **a** Pristine PG, **b** V_C-UP_ C2 vacancy site, **c** V_C-UP_S-doped and **d** V_C-UP_P-doped, respectively. The structures of **c** and **d** as shown in Fig. 3. They are calculated by using PBE functional, the Fermi level is shifted to 0.00 eV

To achieve an in-depth understanding of the nature of C2 vacancy and doping engineering in the HER activity, we investigated the total and projected DOS, electronic band structure of the defect and S-, P-doped PG. Figure 5 is one of our calculation results about electronic band structures, total and projected DOS of pristine PG, V_C-UP_, V_C-UP_S-doped and V_C-UP_P-doped PG.

From the figure, we can see that when C2 vacancy is introduced, some new defect states highlighted by red curves appear in the forbidden band near the Fermi level. Obviously, these new states arise from the C2 vacancy. Furthermore, when S, P heteroatom doping is introduced, the band gap gets narrower (from 2.22 eV [31] to 0.37 eV) and the number of new defect states near the Fermi level increases, which can possibly improve the H* adsorption strength.

However, we found that the DOS of V_C-UP_ near the Fermi level is much larger than that of pristine PG. In addition, the electron density near the Fermi level of C2 vacancy and S-, P-doped PG is further increased relative to pristine PG. We also found that the S 3p and P 3p orbitals undergo significant hybridization with the C1 and C2 states, leading to strong interactions between the heteroatoms and C, and the formation of S–C and P–C bonds. These results demonstrate that combination of the C2 vacancy and S, P heteroatoms doping may be a better engineering for improving HER activity.

#### Electron Density Difference and Charge Transfer

**Fig. 6:**
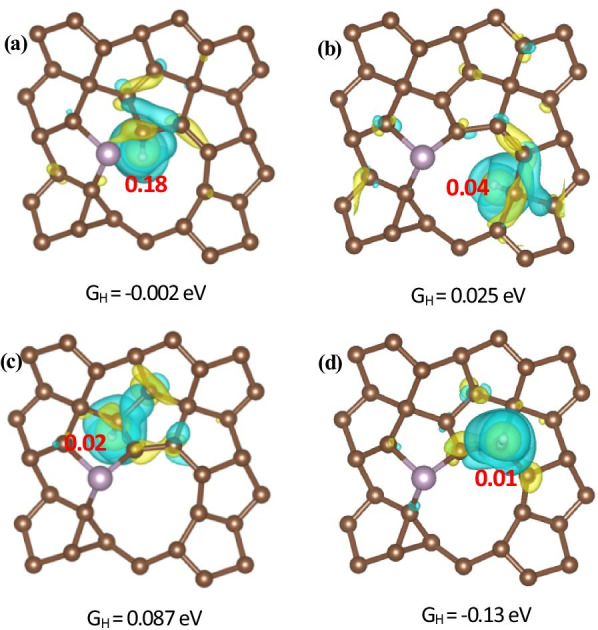
Top views of electron charge density difference and Bader charges analysis for a C_2-DOWN_ vacancy and P-doped PG with H adsorbed at the **a** C1, **b** C_2-DOWN_, **c** C_2-DOWN**n**_ and **d** C_2-UP_ sites. The isosurface level is 0.004 e/Bohr^3^. The yellow and blue colors represent charge accumulation and reduction, respectively

**Fig. 7:**
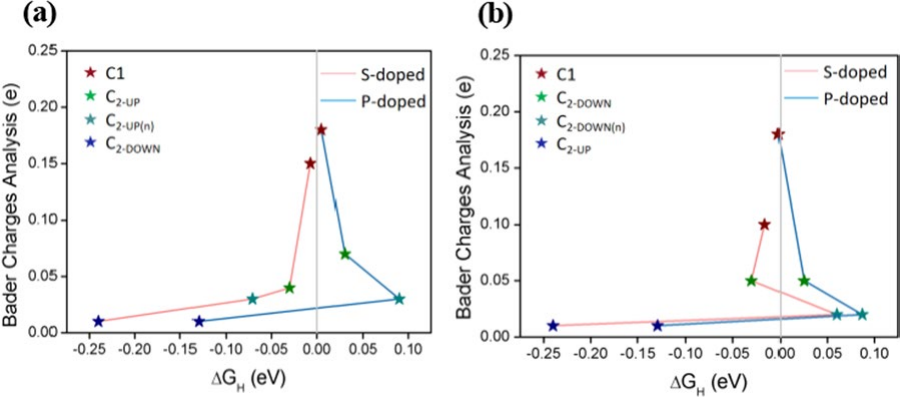
Relationship between Δ*G*_H_ and Bader charges analysis for **a** V_C-UP_S-doped, V_C-UP_P-doped and **b** V_C-DOWN_S-doped, V_C-DOWN_P-doped structures. The Δ*G*_H_ values and active sites are shown in Table 3

Moreover, to study the binding interaction between the H atom and PG, we calculated the electron charge density differences for defect and S-, P-doped PG with different active sites for hydrogen evolution. One of our calculation results about the electron charge density difference and Bader charges analysis for a C_2-DOWN_ vacancy and P-doped PG with H* adsorbed at the different active sites for hydrogen adsorption is shown in Fig. 6. The yellow and blue colors represent charge accumulation and reduction, respectively. It is shown that the electrons accumulate around H atoms and reduce around the C atoms which are bonded to H atoms, indicating a charge transfer from PG to H*. The charge transfer is also confirmed by Bader charges analysis. The calculation results show that there are 0.18, 0.04, 0.02 and 0.01 electrons transferring to H* at C1, C_2-DOWN_, C_2-DOWN(n)_ and C_2-UP_ sites, respectively. We further show that the larger charge transfer from PG to H*, the closer Δ*G*_H_ is to zero, which means the optimal performance for a HER catalyst, as shown in Fig. 7. It can be seen from Fig. 6 that electrons are transferred from PG to H*, resulting in increase in the charge density of the bonds, which means that the stabilization of the H* species in HER performance may originate from the enhanced charge density of P-doped C atoms, indicating that P atoms are inherently advantageous in interacting with H atoms than C atoms. We also noticed that H* is absorbed onto C instead of P, indicating that the increased charge density can contribute to the electrocatalyst on H atom. So our calculations show that P doping into the PG can lead to enhanced adsorption of H* on C atoms. As mentioned above, the DFT calculations also suggested that the P doping into PG could much more efficiently enhance the HER activity than that of S-doping.

### Activity of Defect and Doped PG Toward HER

#### Gibbs Free Energies of HER

**Fig. 8:**
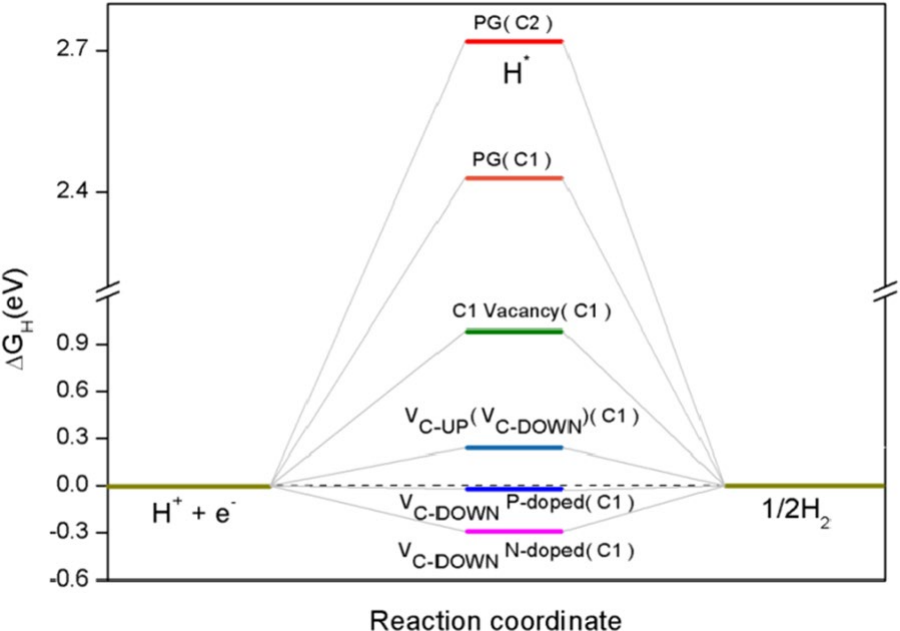
Gibbs free energy versus reaction coordinate of the HER for PG, where C1 and C2 inside the brackets are active sites for hydrogen adsorption

The Δ*G*_H_ is the vital descriptor of the HER for a variety of electrocatalysts, the optimal Δ*G*_H_ value for a electrocatalyst is zero, so the H* adsorption and desorption can occur spontaneously without activation energy barrier [61, 62]. To evaluate the HER activity of the PG and investigate the defection and doping engineering, we calculated the Δ*G*_H_ of HER. One of our calculation results about Δ*G*_H_ versus reaction coordinate of the HER for PG is shown in Fig. 8, where C1 and C2 inside the brackets are active sites for hydrogen adsorption.

Our calculations show that the pristine PG is found to be inert for the HER with a relatively large Gibbs free energy of H* (Δ*G*_H_ = 2.72 eV(C2), Δ*G*_H_ = 2.43 eV(C1)). When vacancies are introduced, there are two different C vacancy sites, C1 vacancy site and C2 vacancy site. We calculated the Δ*G*_H_ on the two sites and found that C2 vacancy can notably decrease Δ*G*_H_ (Δ*G*_H_ = 0.24 eV), which indicates that H* preferentially adsorbs on C2 vacancy structures. The optimized structures with C2 vacancy sites (V_C-UP_ and V_C-DOWN_) are shown in Fig. 2. Though C2 vacancies show significant improvement over the pristine PG, they are still not the optimal for the hydrogen adsorption, so doping engineering is explored to improve the HER performance. We show our effects of C2 vacancies and S, P heteroatom doping on the HER activity and optimize the HER performance. The Δ*G*_H_ values are summarized in Table 3, and the active sites for hydrogen evolution are shown in Fig. 3.

**Table 3:** Calculated Δ*G*_H_ values of C2 vacancies and S-, P-doped PG

Structure	Site	Δ*G*_H_ (eV)
V_C-UP_S-doped	C1	− 0.007
	C_2-UP_	− 0.030
	C_2-UP(n)_	− 0.071
	C_2-DOWN_	− 0.240
V_C-UP_P-doped	C1	0.005
	C_2-UP_	0.030
	C_2-UP(n)_	0.090
	C_2-DOWN_	− 0.130
V_C-DOWN_S-doped	C1	− 0.016
	C_2-DOWN_	− 0.030
	C_2-DOWN(n)_	0.060
	C_2-UP_	− 0.240
V_C-DOWN_P-doped	C1	− 0.002
	C_2-DOWN_	0.025
	C_2-DOWN(n)_	0.087
	C_2-UP_	− 0.130

The calculation results reveal that Δ*G*_H_ decreases significantly, demonstrating that the defection and doping engineering are very effective in reducing Δ*G*_H_. Remarkably, we found that the Δ*G*_H_ values of active sites C1, C_2-UP_ and C_2-UP(n)_ for V_C-UP_, active sites C1, C_2-DOWN_ and C_2-DOWN(n)_ for V_C-DOWN_ are very close to zero, especially for two C1 sites, signifying the optimal conditions can be achieved, which are significantly superior to pristine PG. And we compared our results with previous work from other researchers on graphene, for instance, graphene with C vacancy (Δ*G*_H_ = − 2.108 eV) [28], graphene with N-doped (Δ*G*_H_ = − 0.693 eV) [28], graphene with C vacancy and N-doped (Δ*G*_H_ = − 0.595 eV) [28], graphene with S-doped (Δ*G*_H_ = − 0.30 eV) [29] and graphene with N/S co-doped (Δ*G*_H_ = − 0.12 eV) [29]. We can find that the defection and doping engineering are more effective for PG. Thus, our results clearly suggest that the Δ*G*_H_ of PG can be manipulated by applying defection and doping engineering to achieve the optimal HER activity.

#### The Reaction Pathways of Defect and Doped PG

The HER proceeds in a multistep electrochemical process, via one of two pathways which are known as the Volmer–Tafel and the Volmer–Heyrovsky mechanisms. The first step of HER is the H* adsorption on the electrocatalyst surface (i.e., Volmer reaction), which is described by H^+^  + e^−^  → H*. Then, H* combines with H^+^ and an electron (e^−^) to form a H_2_ molecule, known as the Heyrovsky step, which is described by H* + H^+^  + e^−^  → H_2_. Alternatively, H_2_ molecule can be formed via the Tafel step, i.e., the combination of two H* on the electrocatalyst surface, which is described by 2H* → H_2_ [63].

**Fig. 9:**
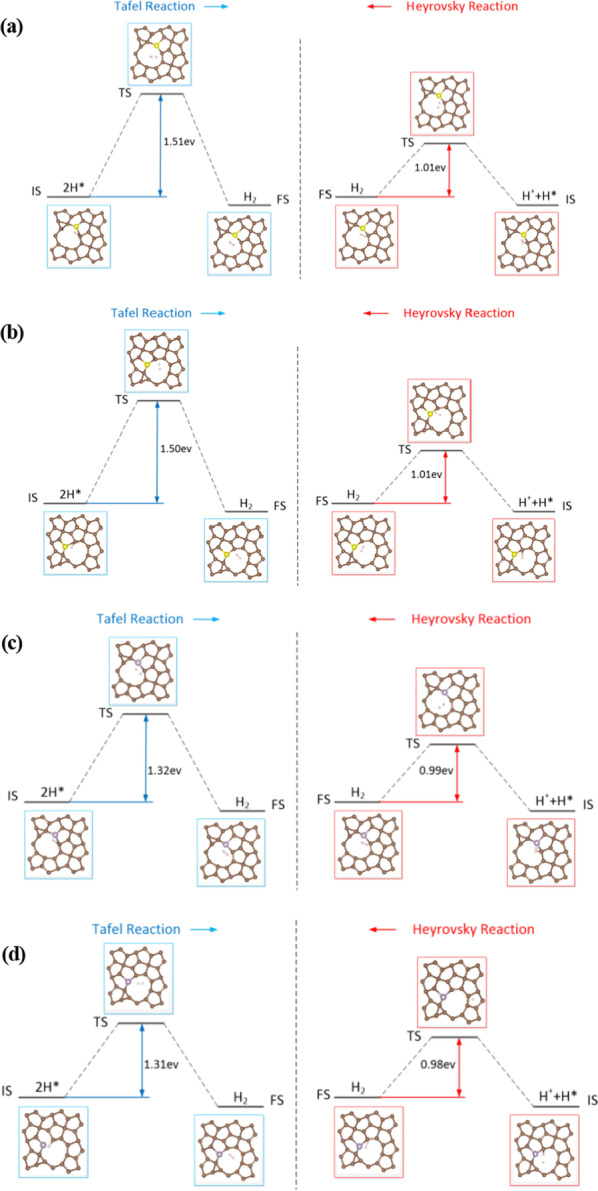
Schematic pathways for the HER. Energy profiles for the Tafel and Heyrovsky reactions with **a** C_2-UP_ vacancy and S-doped PG, **b** C_2-DOWN_ vacancy and S-doped PG, **c** C_2-UP_ vacancy and P-doped PG, **d** C_2-DOWN_ vacancy and P-doped PG. The initial state (IS), the transition state (TS) and the final state (FS) are indicated in the diagram with the corresponding energy barriers

To investigate the defection and doping engineering effects on PG and further understand the mechanism of superior HER activity, the energy barriers of Tafel and Heyrovsky reactions with C_2-UP_ and C_2-DOWN_ vacancies, S-, P-doped PG for C1 site were calculated. The initial state (IS), the final state (FS) and the transition state (TS) are displayed in Fig. 9 with the corresponding energy barriers. For the Tafel reaction, the recombination of 2H* shows energy barriers of 1.51 eV (S-doped), 1.32 eV (P-doped), respectively. Whereas the release of a H_2_ molecule in the Heyrovsky reaction involved in a proton reacting with an adsorbed H* needs to overcome the energy barriers of 1.01 eV (S-doped), 0.99 eV (P-doped), respectively. The results reveal that the energy barriers of Tafel reaction are significantly higher than that of the Heyrovsky reaction. So the HER on defect and doped PG prefers the Volmer–Heyrovsky mechanism.

## Conclusions

We theoretically designed a C vacancy and N-, S-, P-doped PG and investigated their stability and unique role of electrocatalyst toward HER systematically. We find that defection and doping engineering possess a superior HER performance over the pristine PG. Importantly, the optimal HER activity can be achieved with C2 vacancies and S, P heteroatoms doping, which indicates that the catalytic properties of the defect and doped PG can be tuned easily and effectively. Our calculations reveal that Δ*G*_H_ decreases significantly with C2 vacancies and S, P heteroatom doping, and the optimal conditions can be achieved with P doping at C1 active sites, for which defection or doping engineering alone cannot achieve the optimal conditions. The electronic structure analysis shows that when C2 vacancy and S, P heteroatom doping are introduced, several new defection states move closer to the Fermi level, leading to the narrower band gap and an improvement of the hydrogen adsorption strength. We also find the charge transfer from PG to H* by calculating the electron charge density differences, the larger charge transfer to H*, the closer Δ*G*_H_ values to zero by using Bader charges analysis, which indicates the optimal performance for a HER catalyst. And we further demonstrate the HER on defect and doped PG prefers the Volmer–Heyrovsky mechanism. So our study shows that the designed defect and doped PG is highly activated toward HER electrocatalyst, the optimal HER activity can be achieved, and abundant catalytic activity sites are provided. It is expected that the strategies developed in this paper may be applied for designing 2D graphene-based electrocatalysts for low-cost and high-performance HER applications.

## Data Availability

The datasets supporting the conclusions of this article are included within the article, and further information about the data and materials could be made available to the interested party under a motivated request addressed to the corresponding author.

## Abbreviations

HER: Hydrogen evolution reaction
PG: Penta-graphene
2D: Two dimensional
Δ*G*_H_: The Gibbs free energy
TMDs: The transition metal dichalcogenides
VASP: Vienna Ab initio Simulation Package
PAW: Projected augmented wave
PBE: The Perdew–Burke–Ernzerhof
GGA: The generalized gradient approximation
CI-NEB: The climbing image-nudged elastic band
IS: Initial state
FS: The final state
TS: The transition state
